# Intersexual patterns of the digestive tract and body size are opposed in a large bird

**DOI:** 10.1038/s41598-024-66022-z

**Published:** 2024-07-01

**Authors:** Zbigniew Kwieciński, Piotr Tryjanowski, Piotr Zduniak

**Affiliations:** 1 Department of Avian Biology and Ecology, Faculty of Biology, Adam Mickiewicz University in Poznań, Uniwersytetu Poznańskiego 6, 61-614 Poznan, Poland; 2 Department of Zoology, Poznań University of Life Sciences, Wojska Polskiego 71C, 60-625 Poznan, Poland

**Keywords:** Behavioural ecology, Animal behaviour

## Abstract

The appropriate structure of the digestive tract is crucial for individual adaptation to ecological conditions. In birds, the length of the small intestine, responsible for food absorption, is generally believed to be positively correlated with body size. In this study, we investigated the variation in small intestine length in the White Stork (*Ciconia ciconia*), a monomorphic species without visible sexual dimorphism, but characterized by differing parental efforts, which can be reflected by the small intestine lengths between the sexes. We examined the relationship between small intestine length and body size within the sexes. Our findings show that male White Storks have significantly shorter small intestines than females, despite having larger body sizes than the latter. Furthermore, we found a significant relationship between body size and small intestine length, but it was of a different nature in the two sexes. Males exhibited a previously unreported phenomenon, whereby increasing body size was associated with shortening small intestines, whereas females exhibited the opposite pattern. These novel findings shed light on the anatomical adaptations of the digestive tract in birds.

## Introduction

The efficiency of food acquisition and digestion is crucial, as it impacts the foraging behaviour of individuals as well as the depletion rate of food resources^1,2^, and is influenced by many physiological and biological factors^3,4^. Efficient digestion reduces the amount of food needed; inefficient digestion, by contrast, requires more food to meet the organism’s nutritional requirements^1,5–9^. In vertebrates, digestive efficiency depends on the nutrient content of the food, the length of the intestine, which determines the nutrient absorption surface area, and the activity of digestive enzymes^5,10–13^. The small intestine, the longest segment of the digestive tract and responsible for nutrient absorption, is crucial for the efficient digestion of food^10,14,15^. As intestinal tissue is energetically costly to maintain, the size of the intestine should not exceed the organism’s nutritional and energetic requirements^3,16–18^.

Although birds are among the best-known groups of vertebrates in terms of biology and ecology^2,4,19^, there are still knowledge gaps regarding the digestive systems of birds of various body sizes in the context of their feeding and breeding ecology^1,5,6,12,13^. This is mainly because detailed anatomical studies on the digestive system of birds, requiring them to be killed, are difficult to perform under the current ethical standards relating to research on legally protected animal species. Hence, present-day knowledge about avian anatomy comes from the times when ethical standards related to research on animals were of secondary importance.

To date, morphometric studies of the small intestines of passerine birds have revealed different relationships between intestine length, overall body size of the individual and the type and quality of diet. One of them is that a high allometric constant occurs in passerines relating intestine length to body mass. It has been demonstrated that the length of the small intestine increases with increasing overall body size of birds^3^. In turn, the small intestine in herbivorous birds is longer in species that consume more difficult-to-digest or more fibrous food. A long, small intestine can also be expected in carnivorous birds like raptors and storks, which feed almost exclusively on flesh^5,10–12,20^, and omnivorous birds exhibit a high degree of plasticity of the intestinal anatomy depending on the type of food they consume^14,15^. However, experiments were performed to find out whether there were interspecific differences in digestive efficiency and the length of the digestive tract, assessing the possible influence of diet and predatory behaviour on morphological adaptations of the gut^1^. It turned out that predators specializing in fast-moving, agile prey had a relatively short digestive tract. In specialized hunters, such as those that feed primarily on avian prey, adaptation to this one type of food might involve a decrease in overall digestive efficiency and a reduced ability to prey on low-energy food. On the other hand, generalist searchers feeding on relatively slow-moving prey or carrion were found to have long digestive tracts, increasing the efficiency of digestion and allowing them to consume a wide range of prey items, including low-quality ones. This variability recorded in the feeding behaviour and intestinal anatomy of carnivorous birds is associated with different selection pressures^1,5,7,21,22^.

Apart from dietary composition, sex is one of the factors that may govern differences in intestinal anatomy and feeding behaviour within a species. However, differences in intestine length between males and females of particular species have not been investigated. Traditionally, it is believed that species with a high degree of sexual dimorphism differ substantially between the sexes in diet composition^1,5–7^. These differences may result from competitive avoidance, differences in physiology and sex-specific nutrient requirements due to differences in parental effort^23–25^. Sexual dimorphism may also be related to food specialization, with a higher degree of specialization occurring in more dimorphic species^26,27^. However, there is also growing evidence that in species with little or no sexual dimorphism, males and females may differ in several aspects of foraging ecology, such as diet composition, selection of foraging areas and parental feeding^2,28–31^. Thus, studying monomorphic species may shed light on the mechanisms leading to differences in the anatomy of the digestive tract and in parental effort between the sexes.

The White Stork (*Ciconia ciconia*; WS) is a large bird (body mass: 2.3–4.5 kg, wingspan: 155–215 cm) without any distinct sexual dimorphism, where males are c. 12% bigger than females^9,32,33^. The WS is a migratory species that occurs across the whole of Europe except Iceland and the northern regions of Russia and Scandinavia^19^. It builds large, open nests, very often on the top of man-made structures, but less often on trees^31^. The species is characterized by sex-related differences in parental care, with females incubating longer (c. 70%) than males (c. 30%). During incubation periods, males spend c. 55% but females only c. 25% of their time foraging. After the chicks have hatched (up to 10–15 days of age), males provide as much as 80% of the food to the nest^34,35^. The WS diet consists of various small vertebrates and large invertebrates, and the choice of food depends on the seasonal life cycle stage and habitat^36–39^.

The aim of this study was to investigate two main objectives: (1) whether there are differences in the length of the small intestine between the sexes of adult White Storks during the breeding season, and (2) whether there is a relationship within each sex between the length of the small intestine and the size of a stork. We hypothesized that a distinct division of parental effort during different stages of reproduction would be reflected in differences in the length of the small intestine between males and females.

## Results

### Biometrics

Table 1 lists the mean values of basic morphological biometrics of male and female White Storks. We found significant differences between the sexes (t-Student’s test, in all cases *p* < 0.001): males were bigger than females, and the average differences between the sexes ranged from 4.5 to 13.7% (Table 1).

**Table 1:** Biometric measurements of female (n = 51) and male (n = 52) White Storks; mean values are given as the mean with the 95% confidence limits in parentheses.

Trait	Females	Males	Total	Mean difference between sexes (%)
Culmen length (mm)	155.2 (151.4–159.0)	169.7 (166.5–172.9)	162.5 (159.7–165.3)	8.5
Tarsus length (mm)	208.4 (204.1–212.7)	225.5 (221.6–229.5)	217.0 (213.7–220.4)	7.6
Tail length (mm)	228.1 (224.5–231.8)	238.8 (235.9–241.8)	233.5 (231.0–236.1)	4.5
Wing chord length (mm)	551.8 (545.2–558.4)	584.5 (576.7–592.3	568.3 (562.4–574.3)	5.6
Body mass (g)	3076.5 (2954.0–3198.9)	3566.3 (3431.5–3701.1)	3323.8 (3222.1–3425.5)	13.7

### Small intestine length

**Figure 1:**
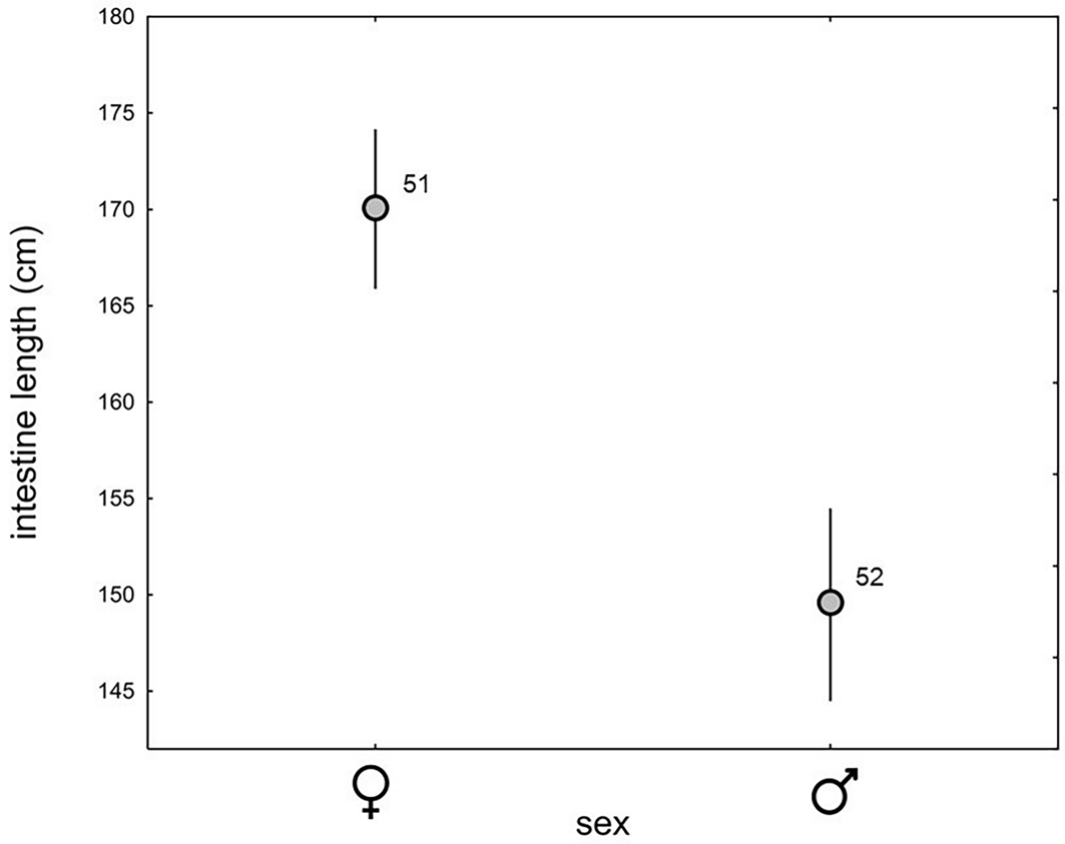
Differences in small intestine length between White Stork females and males; mean values are given with 95% confidence limits; the numbers denote the sample size.

**Figure 2:**
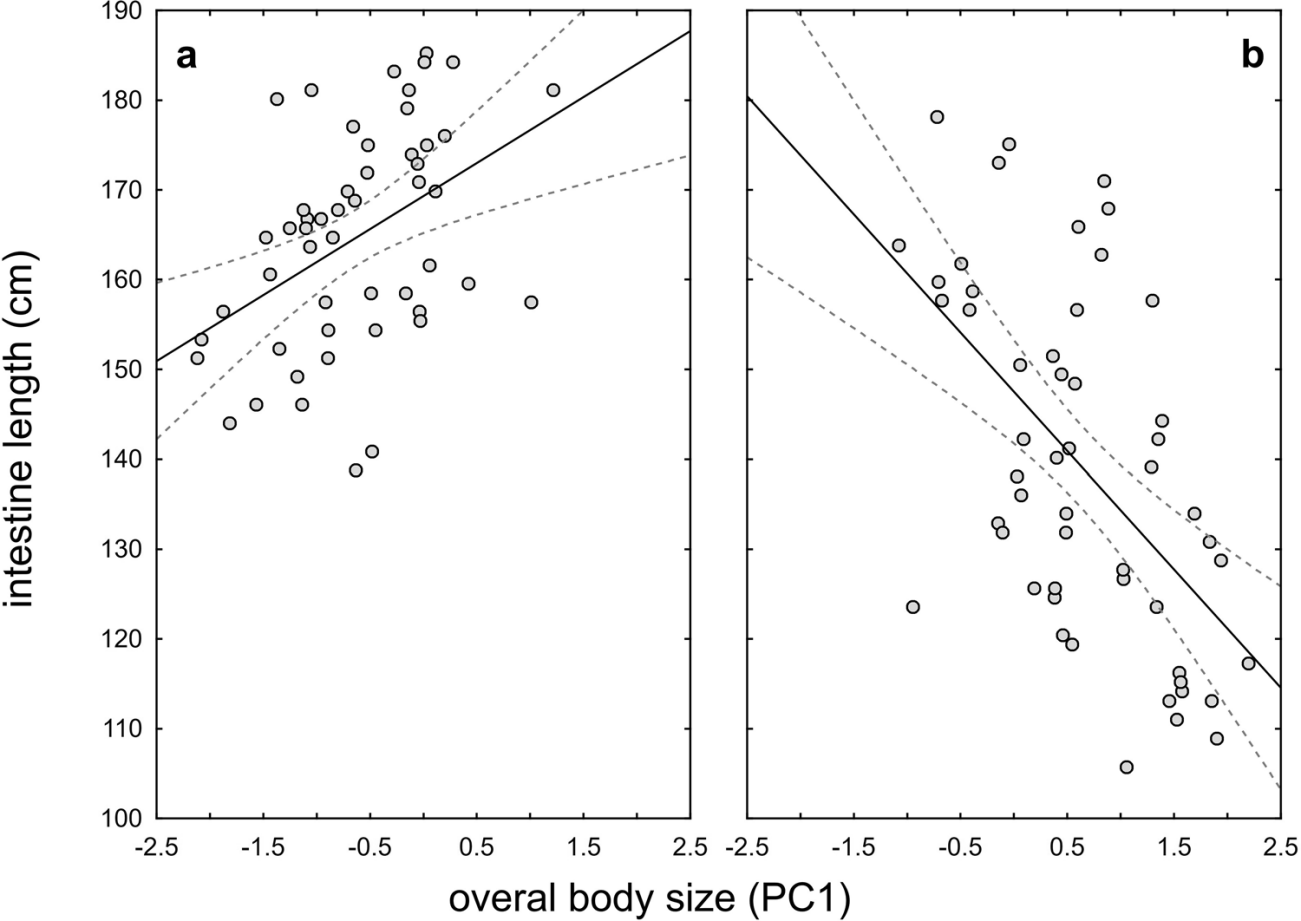
Correlation between small intestine length and overall body size of White Storks according to sex: (**a**)—females, y = 7.36*x + 169.3, R^2^ = 0.19, *p* = 0.001; (**b**)—males, y = − 13.18*x + 147.51, R^2^ = 0.31, *p* < 0.001.

The mean length of the small intestine was 151.9 cm (95% CL 147.8–155.9) and varied between 105.7 and 185.2 cm. We found significant differences between the sexes (GLM, F_1, 99_ = 40.92, *p* < 0.001): the intestines of females were on average 12.0% longer than those of males (Fig. 1). Furthermore, the GLM did not show a significant main effect of the OBS (F_1, 99_ = 0.51, *p* = 0.48) on SIL, but we did find a significant interaction effect of sex and OBS on SIL (sex * OBS: F_1, 99_ = 31.18, *p* < 0.001). The SIL of females increased with increasing OBS (Pearson correlation, r = 0.44; t = 3.39, *p* = 0.001; Fig. 2a), whereas the opposite pattern was found in males (r = − 0.56, t = − 4.75, *p* < 0.001 Fig. 2b).

## Discussion

Our study has shown for the first time that male White Storks have much shorter small intestines than females, but at the same time have a larger body size than females. We also found a relationship between the body size of individuals and the length of the small intestine, but it is different in each sex. In females, the bigger the body size, the longer the small intestine. By contrast, the reverse relationship holds in males, where a larger body size implies a significantly shorter small intestine—this is a new finding in avian anatomical studies. These results suggest that the anatomical differences between the sexes and the inverse relationships within the sexes between body size and small intestine length are influenced by evolutionary differences related to reproduction. These differences likely arise from the differentiation of parental roles and behaviours between the sexes during the pre- and post-natal period, such as nest construction, egg incubation, direct care of chicks, and obtaining and delivering food to the nest. In White Storks, it is the females that primarily incubate the eggs and care for the chicks; hence, they spend more time in the nest than the males. These primarily provide their mates and young with food and defend the nest against other male storks, but increasingly also against White-tailed Eagles *Haliaeetus albicilla*^23,34,40–42^.

The complexity of this revolutionary anatomical-physiological system makes it challenging to separate the influence of diet, flight requirements, parental effort and metabolism of birds when explaining differences in the size of their internal organs without very sophisticated multidisciplinary experimental studies. Our study did demonstrate, however, that the assumption that the length of intestines in birds is directly proportional to the body size of individuals is incorrect. When examining such relationships, it is important to take into account not only interspecific differences, but also sex differences and individual variability within the sexes.

Previous morpho-anatomical studies on birds have shown that the anatomical structure of the small intestine, including its length, volume and number of villi, and also chyme retention, are related to diet, indicating that the intestine adapts to the type and amount of food consumed^43^. For example, insectivorous birds tend to have shorter intestines and villi, as well as thick-walled stomachs, compared to birds with a mixed diet. As an opportunistic feeder, the White Stork exhibits a high digestive efficiency similar to that of carnivorous birds, including piscivorous species, although the structure of its stomach actually resembles that of insectivorous birds^3,44^. Experimental studies on the digestive efficiency and food preferences of male and female White Storks show that digestion is significantly more efficient in females than in males. Moreover, males produce more pellets than females, even though they spend less time foraging than females^8,9^. This suggests that males, with their shorter intestines, transport less digestive content than females, thus regaining their optimal body mass more quickly for further foraging activities; this is an adaptation to ensure optimal body mass for hunting^1,21^. In addition, larger individuals have lower metabolic requirements^21^, which may explain why the males of many bird species are more efficient at catching prey and supply most of the food during the incubation and chick-rearing periods^23,34,40^. Similarly, in male White Storks, it is advantageous to have shorter intestines, as this allows for fast and efficient hunting within a shorter time frame, albeit at the cost of poorer digestion^1,45,46^. Furthermore, during the breeding season, males spend more time outside the nest, so their poorer food digestibility can be compensated for by a longer foraging time and access to a more diverse diet. This is supported by the positive correlation between digestive efficiency and changes in body mass in males, and the opposite, negative relationship in females, which suggests a different feeding pattern between male and female White Storks^8,9,33,44^. Alternatively, the nutrient absorption capacity of the digestive system may be reduced in species with shorter intestines, resulting in longer absorption processes^47^. However, it should be noted that the efficiency of digestion is influenced by the length of the small intestine, as the retention time of ingesta is directly proportional to the length and volume of the intestine. Similarly, female White Storks digest food more efficiently thanks to their longer intestines. This is particularly important during the production of large eggs and the incubation period, when females, on the one hand, spend most of their time in the nest and cannot hunt, and on the other, need sufficient resources and energy to perform their parental tasks^8,9,34,48^. Furthermore, experimental studies have shown that female White Storks have different prey preference patterns than males, being more selective in choosing bird prey while foraging and preferring birds as food to a greater extent^9^. Thus, the preference of female White Storks for bird prey may be an indication of a strategy to rapidly supplement calcium and other nutrients, which are more readily available from avian than from mammalian skeletons^8,49–51^. This could be an important strategy during spring, which requires significant amounts of micronutrients and energy for activities such as egg laying and incubation^52–58^.

## Conclusions

Our study indicates that the elements of bird behaviour related to the reproductive roles of the sexes in particular species are reflected in the anatomy of the digestive system. In the case of the White Stork, where there are sex differences in energy input and parental functions in the pre- and postnatal period, there is a difference in the length of the small intestine between the sexes. But there are also inverse relationships between body size and the length of the small intestine, where the body size of females correlates positively with the length of the small intestine, whereas larger males have shorter intestines than small males. Therefore, when examining the relationship between the length of birds' intestines and body size in species without distinct sexual dimorphism, one needs to take into account not only inter-individual variation, but above all, differences between the sexes.

## Materials and methods

### Study material

During sixteen breeding seasons (May–September 2005–2020), 789 wild White Storks, mainly with broken wings and/or legs, were brought to the rehabilitation centre at the Zoological Garden in Poznań (52°24' N; 16°59' E) by private persons or animal protection services, mainly from the province of Wielkopolska (W Poland). Most of the birds (n = 629; 79.7%) were nestlings that were delivered to the zoo between July and September. Some of the birds had severe bodily injuries that prevented them from functioning either in the wild or in captivity. Therefore, they died or had to be euthanized by intravenous injection (Morbital; dose: 0.6 ml/kg) because of the extent of these injuries (irreversible damage to many organs), so it was they that ultimately made up the study material; this consisted of just 103 adult birds brought to the zoo between May and early July. For our research, we selected only individuals which had been involved in sudden accidents and were present at the rehabilitation centre for a few days at most. The body mass of the birds we examined did not differ from the averages obtained during studies of wild birds from the region^32^.

### Ethical approval statement

The research was conducted under Polish regulations respecting the keeping of animals in captivity and research protocols included in the Animal Protection Act (Dz. U. 1997 Nr 111 poz. 724), with the approval of the Polish National Ethics Committee for Animal Experiments (resolution No. 12/2022), and in accordance with ARRIVE guidelines (https://arriveguidelines.org).

### Morphological biometrics

Fifty-one females and 52 males were measured by one person (Z.K.). Five measurements describing the body size of White Storks^56–59^ were taken: culmen length (a straight line down the centre of the bill from the most distal point to the feathered edge at the base); tarsus length (the metatarsus measured from the proximal to the distal joint of the right leg); wing chord length (from the wrist joint to the tip of the longest primary of the right wing; the left wing was measured if the right wing was injured). The culmen, tarsus and wing chord were measured to the nearest millimetre with a stopped metric ruler. The body mass of each stork was measured with an accuracy of 0.50 g using a Pesola balance (Macro Line 5 kg/50). We selected only adult specimens for the study. The age was determined based on the red colour of the bill and the colour of the metatarsals. Individuals that had any black colouration on parts of the bill and legs were excluded from the study. The sex of the birds was determined at autopsy based on the visible gonads^44^.

### Small intestine length

The intestinal tract was separated post-gizzard adjacent to the pyloric sphincter and at the cloaca, usually within 24 h of the bird’s death. The tract was stripped of fat and mesentery, placed in a physiological position, unstretched in a straight line, and the length measured from the cut-off point at the gizzard to the ileo-cecocolic junction^1^. The small intestine length was measured to the nearest 0.1 cm using a standard ruler according to the method described by Leopold^60^ and DeGolier et al.^61^.

### Statistical analyses

We tested the effect of the sex and overall body size (OBS) of the birds on the small intestine length (SIL) using a General Linear Model (GLM) with SIL as the dependent variable, sex as the categorical variable, and OBS as the continuous dependent variable, including its interaction effect with sex. The OBS of the WS was calculated using Principal Components Analysis (PCA) and was based on several body measurements, i.e. culmen length, tarsus length and wing chord length (all in mm), and body mass (g). The first component (PC1), with initial eigenvalues of 2.59, explained 66.5% of the variation and was highly correlated with all the biometrics included in the analysis (Pearson correlation, range or r coefficients: − 0.70 to − 0.89, n = 103, in all cases *p* < 0.001). The second and the third components (PC2 and PC3) had eigenvalues of < 1 (0.68 and 0.42, respectively) and in accordance with the Kaiser criterion^62^ were not included in the subsequent analyses. PC1 was then transformed by multiplying it by − 1. In this manner, we obtained a variable describing the OBS positively correlated with the biometrics of the individuals studied. The distributions of none of these quantitative variables differed significantly from the normal distribution, except for SIL, the distribution of which was left-skewed (Pearson’s coefficient of skewness = − 1.41; Shapiro–Wilk test, W = 0.96, *p* = 0.002). In the GLM we therefore used the variable transformed for normality by cubing it; the values shown on the graphs were reverse transformed to raw data using cube roots. The calculations were performed using STATISTICA version 13^63^. Throughout the text, mean values are presented with 95% confidence limits (95% CL).

## Data Availability

The datasets used and/or analysed during the current study are available from the corresponding author upon reasonable request.
